# Antiseizure Medications for the Prophylaxis of Migraine during the Anti-CGRP Drugs Era

**DOI:** 10.2174/1570159X21666221228095256

**Published:** 2023-06-15

**Authors:** Eleonora Rollo, Marina Romozzi, Catello Vollono, Paolo Calabresi, Pierangelo Geppetti, Luigi F. Iannone

**Affiliations:** 1 Dipartimento Universitario di Neuroscienze, University Cattolica del Sacro Cuore, Rome, Italy;; 2 Neurofisiopatologia, Dipartimento di Scienze dell’invecchiamento, Neurologiche, Ortopediche e della Testa-Collo, Fondazione Policlinico Universitario Agostino Gemelli IRCCS, Rome, Italy;; 3 Neurologia, Dipartimento di Scienze dell’invecchiamento, Neurologiche, Ortopediche e della Testa-Collo, Fondazione Policlinico Universitario Agostino Gemelli IRCCS, Rome, Italy;; 4 Section of Clinical Pharmacology and Oncology, Department of Health Sciences, University of Florence, Florence, Italy;; 5 Headache Center and Clinical Pharmacology Unit, Careggi University Hospital, Florence, Italy

**Keywords:** Antiseizure-medications, epilepsy, migraine, calcitonin gene-related peptide, glutamate, GABA, pharmacodynamic

## Abstract

Migraine and epilepsy are fundamentally distinct disorders that can frequently coexist in the same patient. These two conditions significantly differ in diagnosis and therapy but share some widely-used preventive treatments. Antiseizure medications (ASMs) are the mainstay of therapy for epilepsy, and about thirty different ASMs are available to date. ASMs are widely prescribed for other neurological and non-neurological conditions, including migraine. However, only topiramate and valproic acid/valproate currently have an indication for migraine prophylaxis supported by high-quality evidence. Although without specifically approved indications and with a low level of evidence or recommendation, several other ASMs are used for migraine prophylaxis. Understanding ASM antimigraine mechanisms, including their ability to affect the pro-migraine calcitonin gene-related peptide (CGRP) signaling pathway and other pathways, may be instrumental in identifying the specific targets of their antimigraine efficacy and may increase awareness of the neurobiological differences between epilepsy and migraine. Several new ASMs are under clinical testing or have been approved for epilepsy in recent years, providing novel potential drugs for migraine prevention to enrich the treatment armamentarium and drugs that inhibit the CGRP pathway.

## 1 INTRODUCTION

Migraine and epilepsy are frequently comorbid disorders, and their association has been recognized for over a century, even before the introduction of electroencephalography [1]. According to a longitudinal 16-year study, lifetime migraine prevalence is 52% greater in people with epilepsy than without. In addition, lifetime epilepsy prevalence is 79% greater in people with migraine than those without [2-4]. A recent meta-analysis reported a 48% estimated prevalence of headache in patients with epilepsy [5]. In line with migraine epidemiology, women with epilepsy reported the occurrence of migraine more often than men. Migraine concomitant with epilepsy increases the risk of developing drug-resistant epilepsy, depression, and anxiety disorders and decreases the patient’s quality of life [6]. Epilepsy and migraine are fundamentally distinct disorders, mandating significant differences in approach to treatment. No relationship between headache type and seizure frequency, epileptic focus location or seizure type has been identified yet [6, 7]. Nevertheless, these two conditions share some widely used preventive treatments. Antiseizure medications (ASMs) are the mainstay of therapy for epilepsy, and about 30 diverse ASMs with different mechanisms of action (MOA) are available to date [8, 9]. Because of their wide spectrum of therapeutic activities, ASMs are among the most often prescribed agents active on the central nervous system (CNS). Indeed, several ASMs are approved treatments in non-epileptic conditions, including migraine, neuropathic pain, bipolar disorder, and generalized anxiety disorder [8, 10]. ASMs may exhibit different tolerability profiles depending on the disease. For example, a higher prevalence of adverse events (AEs) due to ASMs was found in patients with migraine, even though these patients were treated with lower dosages [11].

Topiramate and valproic acid/valproate are the two broad-spectrum drugs approved for treating episodic migraine [12]. Notably, along with onabotulinumtoxinA and monoclonal antibodies against calcitonin gene-related peptide (CGRP) or its receptor (CGRP-R) (anti-CGRP mAbs), topiramate has been approved for the prophylaxis of chronic migraine [13]. High-quality evidence supports the use of topiramate and valproate, which are therefore preferred for a dual purpose in patients comorbid for migraine and epilepsy [14, 15]. Several other ASMs, including lamotrigine and gabapentin, although without approved specific indication, have been tested for migraine in recent decades and are frequently used in migraine treatment [14, 16]. Understanding the mechanisms of ASMs could be useful in identifying the target(s) involved in their antimigraine activity, which may increase our awareness of the neurobiological commonalities and differences between epilepsy and migraine and might be useful in the development of novel migraine therapies [17]. Herein, we review the prophylactic use of ASMs in migraine, including their proposed MOA and clinical evidence of efficacy and tolerability.

## 2 PATHOPHYSIOLOGY AND CLASSIFICATION OVERVIEW

Both migraine and epilepsy are characterized by a common temporal trend, consisting of debilitating attacks separated by interictal periods in a frame of a chronic condition that may last for decades [18]. A recent review detailed the associations between headaches and epilepsy, including information on the pathophysiological mechanisms and genetic variants that link the two disorders [6, 19]. The analysis of the pathophysiology of seizures and migraine headaches underlies some associations and several distinctions between these two disorders.

### 2.1 Pathophysiology: CGRP

Although the pathophysiology of migraine remains mostly unknown, some points of certainty have been acquired in the last four decades by the understanding of the role of the trigeminovascular system as the anatomical and physiological substrate of the episodic abnormal nociceptive transmission underlying the headache and other symptoms of migraine attacks [20]. Recent preclinical and clinical evidence has pointed to the role of CGRP as one of the main migraine mediators. Several RCTs and real-life observational studies have reported the remarkable efficacy and safety of both small molecule CGRP-R antagonists (gepants) and anti-CGRP mAbs in the prophylaxis of episodic and chronic migraine [21]. Gepants are also effective as acute antimigraine medications [22]. Apart from the presence in intrinsic neurons of the gastrointestinal system, CGRP is highly expressed in a subpopulation of C- and Aδ-fiber primary sensory neurons (PSNs) of vagal, dorsal root (DRG), and trigeminal ganglia (TG). In rodents, CGRP is released along with substance P (SP) from peripheral terminals of peptidergic PSNs, thus producing plasma protein extravasation and arteriolar vasodilatation (CGRP), which are collectively referred to as neurogenic inflammation [23].

In humans, there is no evidence supporting the release of SP, whereas CGRP release [23, 24] and its ability to evoke neurogenic vasodilatation [25] have been documented. However, the anatomical site where CGRP exerts its pro-migraine role remains unknown. The negligible penetration of the blood-brain barrier by anti-CGRP mAbs [26, 27] points to a probable peripheral site of CGRP action. Recently, CGRP has been identified [28] as the chemical substance that, as previously proposed by Sir Thomas Lewis in 1936 [29], is liberated by an antidromic invasion of propagated action potentials from PSN terminals to elicit a flare response and to sensitize all neighboring nerve fibers to pain. In mice, CGRP released from periorbital PSN terminals has been found to target its receptor in the surrounding Schwann cells to sustain prolonged allodynia [28]. Thus, the anatomical unit consisting of the varicosity of the peptidergic nerve terminal (from where CGRP is released) and the surrounding Schwann cell (probably of the unmyelinated Remak bundle, which expresses the CGRP-R) and another afferent PSN fiber that conveys the pain signal to the spinal cord may be necessary and sufficient to elicit migraine pain. The observation that human Schwann cells possess the same repertoire of receptors, channels, and enzymes that in mouse Schwann cells sustain persistent mechanical allodynia strengthens the hypothesis of the critical role of the Schwann cell CGRP-R in migraine pain.

In epilepsy, very scarce evidence has been reported for the putative role of CGRP in seizure and epileptogenesis. CGRP seems to facilitate the excitotoxic death of hippocampal neurons in a kainic acid seizure model [30, 31], and a pronounced but transient increase in levels of CGRP was observed after seizures induced by kainic acid but not pentylenetetrazol in several brain areas [32]. Further preclinical and clinical studies are needed to investigate the potential role of CGRP in seizures and epilepsy.

### 2.2 Pathophysiology: CSD

Neuronal excitation/inhibition imbalance is thought to contribute to attack susceptibility in epilepsy and migraine (particularly in migraine with aura) [7, 33]. The possible association between bouts of sensory hyperexcitability and seizures *via* the common contribution of cortical spreading depression (CSD) could be useful in providing a shared mechanistic framework for some clinical features of migraine and epilepsy. CSD was first described in 1944 in a rabbit model of experimental epilepsy [34] and consisted of a self-propagating wave of depolarization that disrupts ionic gradients, associated with a series of local responses, including a transient arterial vasodilation, which is followed by a prolonged (1-2 hours) period of hypoperfusion. As similar findings have been documented by neuroimaging in patients with migraine with aura (but not in migraine without aura) [20, 35], CSD has been proposed as a major pathophysiological mechanism of the aura phase of migraine.

Ionic shifts caused by CSD may release neurotransmitters triggered by variations in intracellular and extracellular potassium sodium, chloride, and calcium ions. Glutamate release plays a leading role in the initiation and propagation of CSD by targeting N-methyl-D-aspartic acid (NMDA) receptors leading to sustained depolarization [36, 37]. Namely, CSD induced a calcium-dependent CGRP release from cortical tissue slices during CSD, and three different CGRP receptor antagonists dose-dependently attenuated CSD, suggesting a critical role of CGRP in this phenomenon [36]. Moreover, CSD transiently opens neuronal pannexin-1 channels [38], which releases inflammatory mediators that dilate intracranial arteries [38].

Although the role of CSD and the ensuing cerebral blood flow changes in the aura and headache phases of the migraine attack remain poorly understood, some studies have shown that the trigeminovascular system, in both its peripheral and central components, is activated by CSD [39]. Induction of CSD by stimulation of the visual cortex in rats increased the activity of central trigeminovascular neurons in the spinal trigeminal nucleus [40] and induced prolonged activation of nociceptors that innervate the meninges [41]. Altogether, these findings support the hypothesis that CSD somehow signals from meningeal afferents to brainstem trigeminal nuclei. However, the precise cellular and molecular mechanisms of the CSD-dependent proalgesic pathway remain to be determined.

Interestingly, experimental seizures in rats have been shown to activate peripheral and central trigeminovascular neurons [42]. In more detail, seizures elicited an initial stimulation of the meningeal nociceptors in the dura [42] that caused selective activation of the different components of the trigeminovascular afferent pathway in the CNS [42]. These findings provided preclinical evidence for a neural pathway implicated in the ictal headache in epilepsy (see below). Various lines of *in vivo* and *in vitro* evidence support the hypothesis that CSD induces CGRP activity in migraine, as recently reviewed [39]. However, current evidence does not seem sufficient, and further preclinical and human studies are needed to establish a cause-and-effect relationship between CSD and CGRP in the migraine mechanism.

### 2.3 Classification and Treatment

Headaches may be associated with epilepsy attacks and are classified, according to their temporal relationship with seizures, into interictal headaches (occurring between > 24 h before and > 72 h after epileptic seizures) and peri-ictal headaches, (occurring shortly before, during, or just after an epileptic seizure) [6]. Therefore, headaches may arise immediately before (preictal), during (ictal), or after (postictal) seizures. In addition to seizure-associated headaches, patients often experience headaches that are not temporally related to seizures. Approximately half of the patients with headaches and epilepsy describe their headaches as severe attacks that markedly affect their quality of life [43]. Accurate classification of epilepsy and headache requires a thorough description of the symptoms and their temporal relations and is essential for initiating appropriate treatments. Whereas the International League Against Epilepsy (ILAE) seizure classification [44] does not include a class of seizures with symptoms that overlap with headaches, the International Classification of Headache Disorders (ICHD-3) [45] considers several categories of seizure-related headaches. These include 1.4.4 migraine aura-triggered seizure; 7.6.1 Ictal epileptic headache; 7.6.1 Hemicrania epileptica; and 7.6.2 Post Ictal Headache [45]. Currently, there are no published guidelines on managing headaches in people with epilepsy.

**Fig. (1):**
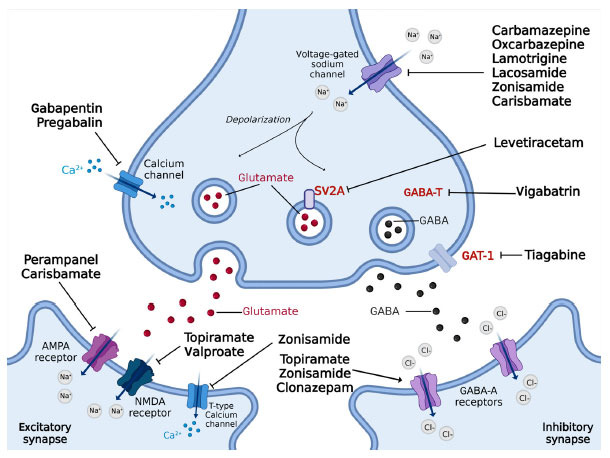
Main pharmacological targets of ASMs used in migraine prophylaxis. **Abbreviations**: AMPA, alpha-amino-3-hydroxy-5-methyl-4-isoxazolepropionic acid; GABA, gamma-aminobutyric acid: GABA-T, GABA transaminase; GAT-1, GABA transporter-1; NMDA, N-methyl-D-aspartic acid; SV2A, synaptic vesicle glycoprotein 2A.

Numerous ASMs, the mainstay of therapy for epilepsy, have been tested for migraine prophylaxis by RCTs and open-label studies (Table **1**), with very different and sometimes inconsistent results. Only two ASMs, sodium valproate/ divalproex sodium and topiramate, characterized by a large spectrum of MOAs, are currently approved for migraine prophylaxis and epilepsy. The specific mechanism(s) responsible for their antimigraine efficacy is still unknown. It is broadly supposed that the role of ASMs in migraine prevention lies in their potential to block CSD and prevent central sensitization [46]. Generally, ASMs act by stabilizing neuronal membranes through their effect on voltage and receptor-gated ion channels and reducing the release of pro-algesic neuropeptides (Fig. **1**) [47]. Recent evidence (see paragraphs on ASMs) suggests that ASMs might exert their antimigraine activity by attenuating neurogenic inflammation in the trigeminal vascular system, directly in the release of CGRP, or by several additional MOAs in the central and peripheral nervous system (CNS and PNS, respectively).

## 3 ANTISEIZURE MEDICATIONS ASSESSED FOR MIGRAINE TREATMENT

### 3.1 Topiramate

Topiramate is a structural analogue of fructose-1,6-diphosphate, synthesized initially as a gluconeogenesis inhibitor, even though it failed to show any anti-diabetic activity [48]. Due to the structural similarity of a sulfamate portion to sulfonamide anticonvulsants, it was evaluated for a possible anticonvulsant effect and revealed to be highly effective in both models of generalized and drug-resistant focal epilepsy [49, 50]. Topiramate is a broad-spectrum neuroactive drug, and several different MOAs have been identified and linked to its broad anticonvulsant effect. It negatively modulates voltage-sensitive sodium channels in a concentration-dependent manner [51, 52]. Moreover, it inhibits alpha-amino-3-hydroxy-5-methyl-4-isoxazolepropionic acid (AMPA) and kainate subtypes of glutamate receptors [53, 54] and negatively modulates high voltage-activated calcium channels [55, 56]. Topiramate enhances gamma-aminobutyric acid (GABA)-A mediated inhibition by increasing the frequency of the chloride channel opening [57] and by enhancing synaptic GABA release [58]. Finally, it shows carbonic anhydrase inhibiting properties, which are thought to activate a hyperpolarizing potassium conductance [59]. Topiramate is indicated as monotherapy or adjunctive therapy for focal epilepsy and generalized epilepsy, including Lennox-Gastaut syndrome [60]. Moreover, topiramate 50-100 mg is indicated as a first-line agent for episodic and chronic migraine prophylaxis, with a level A recommendation [61].

Several mechanisms have been suggested to explain topiramate efficacy in migraine prophylaxis. Topiramate acts centrally on pain activation and sensitization mechanisms through its broad pharmacodynamic properties targeting multiple channels and neurotransmitter functions [62]. First, topiramate modulates cortical hyperexcitability, increasing the threshold for induction and reducing the progression of CSD through its inhibitory GABA-mediated effect and inhibition of Na^+^ and Ca^2+^ conductance [12, 63]. Topiramate attenuated CSD in rats in an experimental setting where CSD elicited CGRP release [36]. Moreover, its modification of the excitatory transmission mediated by AMPA and kainate receptors, coupled with the negative modulatory effect on Na^+^ and Ca^2+^ currents, is thought to decrease or prevent the release of neurotransmitters and neuropeptides involved in the central pain pathway, such as the caudal trigeminal nucleus [63]. Such effects were demonstrated in animal models, in which topiramate inhibited the activity of trigeminovascular nociceptive neurons, and subsequent neurogenic dural vasodilatation, through glutamatergic and GABAergic pathways [64].

Topiramate was first investigated for migraine prophylaxis in a double-blind, randomized, placebo-controlled trial involving 40 patients diagnosed with episodic migraine with aura and without aura [65]. Topiramate was effective and well tolerated, with a significantly greater reduction in the monthly migraine rate than the placebo. Subsequently, three larger, multicenter, double-blind RCTs robustly established topiramate efficacy in migraine prophylaxis [66-68]. These three trials investigated 50-200 mg topiramate in episodic migraine with and without aura in a 6 month-treatment, assessing the change in mean monthly migraine frequency from baseline through the entire double-blind treatment period. The change from baseline in the monthly migraine frequency was statistically significant for patients treated with topiramate at either 100 mg or 200 mg compared with placebo, but not in the arm of topiramate 50 mg. The responder rates (*i.e*., patients with at least a 50% reduction of monthly migraine frequency) were 39% (50 mg), 49% (100 mg), and 47% (200 mg) *vs*. 23% with placebo [67]. A second study [66] confirmed the efficacy of reducing migraine frequency from a baseline of 100 or 200 mg topiramate. More topiramate-treated patients (50 mg/day, 35.9%; 100 mg/day, 54.0%; and 200 mg/day, 52.3%) exhibited at least a 50% reduction in monthly migraine frequency than placebo-treated patients (22.6%) [66]. The third trial [68] compared topiramate 100 mg or 200 mg, propranolol 160 mg, and placebo. Patients treated with topiramate 100 mg had a significantly greater reduction in monthly migraine frequency compared with placebo. In contrast, patients treated with topiramate 200 mg did not, probably due to the high rate of dropouts in this treatment arm.

The topiramate 100 mg and propranolol groups were similar in migraine frequency reductions, responder rate, and medication use.

A large, randomized, double-blind, placebo-controlled trial enrolling 306 patients for 16 weeks demonstrated topiramate 100 mg efficacy in the prophylaxis of patients with chronic migraine [69]. This trial also investigated the impact of migraine-related disability, showing a significant and clinically meaningful improvement in the ability to perform daily activities. Other smaller RCTs confirmed the efficacy of topiramate in CM [70-72].

Another open-label RCT evaluated topiramate 75 mg and valproate 750 mg in 44 patients with chronic migraine [73], showing intra-group similar significant improvements in monthly migraine frequency for both drugs. A large, double-blind, placebo-controlled RCT (the PROMPT trial) revealed a sustained benefit after discontinuing a 6-month topiramate treatment during a double-blind phase of 26 weeks, as the number of migraine attacks did not return to its original levels. However, they did increase in the first 4 weeks after drug withdrawal [74].

Topiramate efficacy in migraine prophylaxis was also evaluated in comparative studies with other oral preventive drugs. However, none of these studies had a superiority design, and most did not show a significant difference between topiramate and the other drugs. In a multicenter, double-blind RCT, topiramate up to 100 mg daily was compared with amitriptyline 50-100 mg in patients with episodic migraine with and without aura, revealing no statistically significant difference between the two drugs (least square mean change from baseline in the monthly rate of migraine episodes: -2.6 in the topiramate group *vs*. -2.7 in the amitriptyline group) [75]. In a double-blind, cross-over RCT, topiramate 25 mg was compared with lamotrigine 25 mg, revealing no efficacy nor tolerability difference between treatments in episodic migraine [76]. In a double-blind RCT, a total of 126 episodic migraine patients were randomized to receive either topiramate 100 mg, flunarizine 5 mg, or both drugs for 12 months, revealing no significant differences among the three groups [77]. In two RCTs, topiramate 50 mg was found to be equivalent to sodium valproate 400 mg [78, 79] in terms of efficacy and safety. Finally, topiramate 50 mg was more effective than propranolol 80 mg in a double-blind RCT of patients with episodic migraine [80]. However, such results should be taken cautiously due to the small sample size of both trials.

Recently, a randomized, double-blind, controlled trial was performed to compare the tolerability (primary outcome discontinuation due to adverse events) and efficacy of erenumab *vs.* topiramate (50-100 mg/day) for migraine prophylaxis (HER-MES study) [81]. Whereas only 10.6% of patients discontinued erenumab, 38.9% discontinued topiramate due to adverse events. Moreover, more patients achieved a ≥50% reduction in migraine days per month with erenumab (55.4%) than topiramate (31.2%) [81]. Overall, erenumab demonstrated a better safety and efficacy profile compared to topiramate.

The most common side effects of topiramate reported in the RCTs were paresthesia, anorexia, weight loss, altered taste, memory impairment, fatigue, insomnia, nausea, diarrhea, somnolence, and dizziness [66-68]. These side effects were dose-dependent; generally, the tolerability of 100 mg/day was better than 200 mg/day. However, a pooled analysis of the three RCTs of topiramate *versus* placebo [82] showed that adverse events other than paresthesia and any cognitive symptoms led to drug discontinuation in fewer than 5% of the patients. Topiramate is associated with a distinct cognitive syndrome involving word-finding problems, slowed thinking/mental processing, attention and memory difficulties [62], and psychiatric/behavior disturbances, such as depression and mood problems [83]. Notably, neurocognitive symptoms and paresthesia are more prominent in patients treated with topiramate for migraine than epilepsy, suggesting a possible underlying disorder-dependent vulnerability [84]. Moreover, treatment with topiramate is associated with a 10-fold increased risk of nephrolithiasis due to increased urinary bicarbonate excretion and urinary pH and a reduction in urinary citrate [85]. Topiramate 50-100 mg is a first-line agent for migraine prophylaxis, with high-level evidence in chronic migraine and medication overuse headaches. Topiramate is generally considered safe and well-tolerated in migraine treatment, but valproate is usually characterized by low adherence [86, 87]. However, it requires careful monitoring of the patient with regard to cognitive problems, language disturbances [88], and behavior disturbances, including a rare risk of suicidal ideation [89], risk of nephrolithiasis, and weight loss. Finally, women of childbearing potential should be cautioned regarding topiramate's possible teratogenicity [83].

### 3.2 Valproate, Sodium Valproate, or a Combination of the Two (Divalproex Sodium)

Valproate (VPA, 2-propylpentanoic acid) was first synthesized in 1882 as an analogue of valeric acid and serendipitously discovered as an anticonvulsant in 1962 [8]. Valproate, sodium valproate, or divalproex sodium (a mixture of the two) have indications in Europe and the US for epilepsy, bipolar disorders, and migraine (with a level A recommendation for the American headache society guidelines) [61]. For epilepsy, valproate is indicated as monotherapy or adjunctive therapy for focal epilepsy and generalized epilepsy, including Lennox-Gastaut and Dravet syndromes.

The MOAs of valproate include several different targets: blockage of voltage-gated sodium channels and T-type calcium channels, enhancement of GABA neurotransmission, increase of potassium conductance, inhibition of NMDA-evoked neuroexcitatory signals, turning off of 5-HT neurons of the dorsal raphe nucleus, and reduced activation of the trigeminal nucleus caudalis [8, 12]. Furthermore, valproate has been demonstrated to act peripherally, reducing neurogenic inflammatory responses in the trigeminal vascular, apparently mediated by GABA-A receptor agonism [16]. Overall, valproate increases neuro-inhibitory GABA activity by inhibiting GABA transaminase and succinic semialdehyde dehydrogenase, enhancing the postsynaptic response to GABA. Valproate can also directly act on GABA receptors, enhancing stimulus-induced responses at both GABA-A and GABA-B receptors [90]. Finally, valproate can act through epigenetic mechanisms, mainly depending on the inhibition of the histone deacetylase (HDAC) and the modulation of brain-derived neurotrophic factor (BDNF) [90]. Notably, active metabolites (2-en-valproic acid) can accumulate, yielding concentrations that may activate glutamic acid decarboxylase, which synthesizes GABA. High doses could also increase extracellular 5-HT and dopamine in the hippocampus and striatum [8, 12].

Despite early attempts to correlate specific MOAs of valproate with putative migraine mechanisms, the cellular and molecular pathways implicated in the preventive effect of migraine of valproate are still unclear [14, 91]. Among the several MOAs of valproate, the influence on GABA/ Glutamate neurotransmission, reduction in trigeminal nucleus caudalis activity, and the GABA receptor-dependent switch-off of 5-HT neurons in the dorsal raphe nucleus are those with the more robust preclinical evidence. Notably, all ASMs used in migraine share the common mechanism to enhance GABA-mediated inhibition. However, drugs acting directly on GABA systems are ineffective in migraine treatment [92, 93].

Valproate has established efficacy as a prophylactic treatment of migraine based on open-label studies and RCTs, favoring doses ranging from 500 to 1000 mg/day [90, 94-97]. Among several trials, five studies provided compelling evidence for the efficacy of divalproex sodium [98-100] and sodium valproate [101, 102]. Valproate was compared with placebo and/or other active interventions, but no RCTs have been performed comparing valproate with other ASMs. In particular, five trials compared valproate with a placebo, five with another active intervention (two cinnarizine and three topiramate), and two compared valproate with both placebo and propranolol or levetiracetam [103]. Overall, pooled analysis suggests that a 50% reduction in headache frequency is twice as likely in patients taking divalproex sodium and three times as likely in patients taking sodium valproate, compared with placebo [16, 94]. Compared with other migraine-preventive drugs in open or single-blind trials, valproate is at least equally effective as propranolol and equally or slightly less effective as topiramate in migraine prevention [16, 94]. Overall, valproate demonstrated low adherence, similar to other ASMs and oral preventive drugs [86, 87].

In trials for migraine prevention, the most clinically significant AEs were fatigue, weight gain, dizziness, nausea, and tremor. However, patients should still be monitored for severe, although rare, AEs, including liver failure and pancreatitis. The use of valproate in migraine is declining due to its demonstrated teratogenicity. The Food and Drug Administration/European Medicine Agency (FDA/EMA) recommends avoiding its use in women with childbearing potential. During pregnancy, valproate (class X) is contraindicated to prevent the major risk of congenital malformations and neurodevelopmental disorders. Valproate should be avoided in liver-compromised patients [8].

### 3.3 Zonisamide

Zonisamide (ZNS) is a second-generation sulfonamide anticonvulsant introduced in 1972 with a unique chemistry among ASMs, consisting of aromatic fused 12 benzene-isoxazole rings and a sulfonamide side chain. Zonisamide and topiramate are the only sulfur-containing ASMs, and, in addition to their similar chemistry, they inhibit carbonic anhydrase and show some analogy in AEs (weight loss). Zonisamide is currently approved as an add-on therapy for focal seizures in adults. Still, it does not indicate migraine or other headaches, although some reports show its use in migraine prophylaxis.

Zonisamide has several MOAs that are partially similar to topiramate and include a reduction in voltage-gated sodium channels and a unique mechanism among ASMs, low-voltage-gated T-type calcium channels [8]. These calcium channel subtypes have been implicated in mediating the release of CGRP in the trigeminal ganglion and nucleus caudalis [104]. Other MOAs include the modulation of GABAergic enhanced GABA release, a phenomenon that results in the downregulation of the number of GABA reuptake transporter proteins and glutamatergic neurotransmission associated with upregulating the glutamate transporter EAAC-1; increase in dopaminergic and serotonergic transmission by enhancing extracellular levels of these monoamines; partial inhibition of carbonic anhydrase. As for topiramate, inhibition of the carbonic anhydrases reduces the buffering properties of the HCO^−^_3_ /CO_2_ system, leading to acidosis. However, this mechanism is unlikely to be relevant in migraine prophylaxis; more potent carbonic anhydrase inhibitors, such as acetazolamide, have minimal antimigraine activity. No pharmacological evidence has yet been reported that carbonic anhydrase inhibition is responsible for the anticonvulsant activity of zonisamide. Finally, zonisamide demonstrated centrally mediated anti-hyperalgesic and anti-allodynic effects, partly *via* the blockade of nitric oxide (NO) synthesis in a mouse model of partial nerve injury. [10]. Overall, zonisamide has a complex action of releasing several neurotransmitters, including GABA, dopamine, serotonin, and acetylcholine. How these effects could contribute to the antimigraine activity of zonisamide is uncertain.

There is only one single-center, randomized, placebo-controlled study comparing zonisamide (50 to 200 mg/day) and topiramate (25 to 100 mg/day) [105] that showed that ASMs are efficacious in episodic migraine, reducing headache frequency, severity, and analgesics intake. In two open-label, uncontrolled, small studies, zonisamide decreased the number of headache days and migraine severity compared to the placebo [106, 107]. In another small open-label study, zonisamide was safe and effective in patients who previously had not tolerated topiramate [108].

Common AEs include fatigue, paresthesia, kidney stones, memory impairment, decreased hemoglobin level, and weight loss [109]. Visual side effects such as palinopsia may occur [110]. Women of childbearing age should be cautioned regarding teratogenicity. Clinically, zonisamide should be considered an alternative ASM in patients who cannot tolerate topiramate [106-109]. However, large RCTs are still needed to determine conclusive evidence of its use in migraine.

### 3.4 Lamotrigine

Lamotrigine is a phenyltriazine derivative that differs chemically from other types of ASMs. Lamotrigine selectively binds and inhibits voltage-gated sodium channels through a voltage- and frequency-dependent blockade and, consequently, inhibits the release of excitatory amino acids, glutamate, and aspartate. It exhibits an additional inhibitory activity on voltage-gated calcium channels [111-114]. In adults, lamotrigine is approved as adjunctive or monotherapy treatment for focal-onset seizures, generalized seizures, tonic-clonic seizures, and Lennox-Gastaut syndrome. Lamotrigine is also indicated for the treatment of bipolar I disorder. Off-label uses of lamotrigine include the treatment of chronic pain with controversial results, unipolar depression, schizoaffective disorder, and borderline personality disorder [115, 116]. The reduction in intracellular calcium levels by lamotrigine during paroxysmal depolarizing events and, perhaps, during CSD could explain the efficacy of lamotrigine in migraine with aura. At the same time, the release of glutamate may be essential in the propagation of CSD [12]. Lamotrigine has been studied mostly in the prophylaxis of migraine with aura. Lamotrigine is established as ineffective for migraine prevention according to the guidelines of the American Headache Society [61].

A randomized, double-blind trial with lamotrigine in 77 patients with migraine with or without aura showed no significant benefit of lamotrigine in reducing the attack rate compared to a placebo. It should be noted that, in the lamotrigine group, only 14 patients reported migraines with aura attacks, which could explain the negative result. Moreover, lamotrigine at 200 mg/day was associated with a higher frequency of skin rashes [117]. In an open study of 44 patients with migraine with aura (including patients with brainstem aura, hemiplegic migraine, and aura without headache) who had been treated with lamotrigine ranging from 50 mg/day to 200 mg/day, thirty (68%) patients reported a beneficial effect [118]. A controlled prospective open study on a cohort of patients suffering from migraine with aura showed that among the 44 responders, lamotrigine (range 50-300 mg/day) reduced the duration of aura symptoms and decreased the mean frequency of migraine attacks [119]. In an open-label study on 21 patients affected by migraine with aura with lamotrigine 100 mg/day, the mean frequency per month of migraine with aura attack was reduced from 6.1 ± 4.1 to 0.7 ± 1.3 over 3 months. Thirteen patients did not report any further attacks of migraine with aura [120]. Another small open study confirmed the efficacy of lamotrigine in reducing aura episodes and duration [121].

Lamotrigine has also been effective as a preventive treatment in more severe types of aura, such as brainstem aura and hemiplegic migraine [122-124]. Lamotrigine may also be effective as a prophylactic treatment for short-lasting, unilateral, neuralgiform headache attacks with conjunctival injection, tearing, and rhinorrhea (SUNCT syndrome) [125]. Cutaneous adverse events are common adverse effects of lamotrigine (including the uncommon Stevens-Johnson syndrome). Cutaneous adverse reactions reported in the clinical trials of lamotrigine in the prophylaxis of migraine were mainly mild maculopapular rashes. A slower titration decreased the risk of serious cutaneous reactions. Other common adverse effects include dizziness, vertigo, sleep disturbances, weight increase, and asthenia [117, 120, 121]. Therefore, lamotrigine seems to be an effective preventive treatment for migraines with aura, including more severe auras. In contrast, its efficacy for migraine without aura is still a matter of debate. Lamotrigine was associated with a high risk of cutaneous rash, especially with a fast titration, which often led to the discontinuation of the therapy in clinical trials. Overall, the limitations of lamotrigine use in clinical practice are its need for a slow titration and the clinically significant drug-drug interactions. Finally, it should be noted that contrariwise to the majority of ASMs, lamotrigine can be used during pregnancy.

### 3.5 Levetiracetam

Levetiracetam is a broad-spectrum second-generation antiseizure medication [126]. Levetiracetam is a pyrroline derivative structurally unrelated to any other antiepileptic drug. The most relevant MOA is binding to the synaptic vesicle glycoprotein 2A (SV2A) [126]. SV2A is ubiquitously expressed in the CNS. The specific function of SV2A is largely unknown, but it plays a role in vesicle exocytosis and the modulation of synaptic transmission, resulting in reduced neuronal hyperexcitability [127, 128]. Levetiracetam has also been shown to indirectly affect GABAergic neurotransmission and inhibit N-type calcium channels [129, 130]. Currently, levetiracetam has been approved for the treatment of focal onset seizures with or without secondary generalization in adults, adolescents, children, and infants from 1 month of age with epilepsy, in the treatment of myoclonic seizures in adults and adolescents from 12 years of age with juvenile myoclonic epilepsy and the treatment of primary generalized tonic-clonic seizures in adults and adolescents from 12 years of age with idiopathic generalized epilepsy. Levetiracetam has also been studied in treating neuropathic pain, tardive dyskinesia, and Huntington's disease [131, 132]. It is not approved for migraine or other headaches.

The therapeutic action of levetiracetam in migraine could be explained by the inhibition of N-type calcium channels and its possible effect on CSD [133, 134]. Another mechanism of action of levetiracetam could be related to its modulatory effect on the GABAergic system [135]. A prospective randomized placebo-controlled study in 52 patients with episodic migraine with or without aura demonstrated that patients treated with levetiracetam (1000 mg/day) significantly reduced the frequency and severity of migraine compared to the placebo group [136]. A prospective RCT on 85 non-treated patients with episodic migraine, randomized to receive levetiracetam 500 mg/day, valproate 500 mg/day, or placebo showed a beneficial effect of both drugs when compared to placebo, which, however, did not differ significantly when compared to each other [137].

Another single-center open-label study in a small cohort of patients affected by migraine with aura showed that levetiracetam (1000 mg/day) significantly reduced the number of attacks per month. This effect improved over the first 3 months of treatment and remained unchanged until 6 months. Moreover, by the third month of treatment, the aura had disappeared entirely in 7 patients [138]. In another open-label study, levetiracetam was well tolerated in 13 elderly patients with migraine. The monthly frequency of attacks was 12.2 ± 5.9 at baseline, 8.3 ± 4.9 after one month, 4.1 ± 2.6 after three months, and 1.3 ± 1.4 after six months with a significant reduction compared with basal values [139].

Levetiracetam has been studied in chronic migraine in a prospective, open-label study with 36 patients at doses ranging from 1000 to 3000 mg/day. Headache frequency was significantly decreased from baseline (26.1 days/month) to the end of the study (14.3 days/month) [140]. A placebo-controlled, crossover RCT enrolled 89 patients with chronic daily headache (CDH) who received levetiracetam (target dose of 3000 mg/day) and 88 who received a placebo. Levetiracetam achieved a 3.9% increased headache-free rate over the placebo, but the difference was not statistically significant. However, 10% of the patients lost the diagnostic criteria for CDH, and in the levetiracetam group, patients experienced decreased attacks and disability [141].

Levetiracetam exhibited an overall good tolerability profile in the prophylaxis of migraine, the most frequent adverse events include somnolence, dizziness, irritability, anxiety, weight gain, depression, asthenia, memory problems, and lack of concentration [142]. Levetiracetam demonstrated moderate levels of efficacy as a prophylactic treatment for patients with migraine, although sometimes a significant difference with a placebo was not obtained. Although levetiracetam requires renal function monitoring, its administration may require dose adjustment for patients with renal impairment [126]. Levetiracetam has a favorable pharmacokinetic profile, excellent bioavailability, minimal plasma protein binding, quick achievement of steady-state concentrations, and no clinically relevant drug-drug interactions.

### 3.6 Gabapentin/Pregabalin

Gabapentin and pregabalin are structurally related to the neurotransmitter GABA and both act on GABA-mediated neurotransmissions, although they do not affect GABA receptors. These two drugs have a high affinity for voltage-gated calcium channels, especially for the subunit α2δ‐1, reducing the release of excitatory neurotransmitters such as glutamate [143]. The mechanism of these ASMs in migraine prevention may be mediated by their action on calcium channels, reducing the calcium influx and, subsequently, the synaptic release of several excitatory neurotransmitters, such as glutamate. Moreover, they may act through the modulation of GABA-mediated transmission [12]. Gabapentin and pregabalin may also reduce the release of inflammatory neuropeptides that cause headache pain, such as CGRP and substance P [144]. Gabapentin and pregabalin are overall well tolerated. The most frequent adverse events reported in the treatment of migraine are asthenia, nausea, somnolence, confusion, dizziness, infections, ataxia, weight gain, blurred vision, drowsiness, abnormal thinking, and constipation [145-148].

#### 3.6.1 Gabapentin

Gabapentin is approved for managing peripheral neuropathic pain in adults and as adjunctive therapy in treating focal onset seizures, with and without secondary generalization in adults and children aged 6 years and over. Gabapentin is also indicated as a monotherapy in the treatment of focal seizures with and without secondary generalization in adults and adolescents. Gabapentin has a level U of evidence for its use in migraine prophylaxis [61]. Gabapentin was found to be effective in a double-blind, placebo-controlled trial for migraine prophylaxis in patients with or without aura (56 patients) over placebo (31 patients) at a stable dose of 2400 mg/day, obtaining a significant reduction in 4-week migraine rate (2.7 for gabapentin- and 3.5 for placebo-treated patients respectively, with gabapentin maintained on a stable dose of 2400 mg/day -) [145].

A double-blind RCT on 63 patients affected by migraine with or without aura with GBP titrated up to 1200 mg/day demonstrated a significant reduction in the frequency and intensity of headache in 47.6% of gabapentin-treated patients [149]. In a double-blind RCT with gabapentin 2400 mg/day on 95 patients with CDH, the percentage of headache-free days per month showed a 9.1% difference favoring gabapentin [148]. The results of open-label studies were consistent with the efficacy of gabapentin for migraine prophylaxis [150, 151]. Conversely, one double-blind RCT failed to demonstrate a statistically significant benefit of gabapentin enacarbil titrated to 1200 mg/day (66 patients), 1800 mg/day (134 patients), 2400 mg/day (133 patients), or 3000 mg/day (62 patients), over placebo (128 patients) in migraine prophylaxis [152].

#### 3.6.2 Pregabalin

Pregabalin is approved for treating neuropathic pain, as adjunctive therapy in adults with focal seizures with or without secondary generalization, and for generalized anxiety disorder. In a double-blinded RCT on episodic migraine, pregabalin (42 patients) and valproate sodium (46 patients) had comparable efficacy in reducing migraine frequency, intensity, and duration of attacks [153].

In an open-label study on thirty patients affected by chronic migraine, pregabalin treatment was associated with a significant decrease in headache frequency, severity, and intake of symptomatic drugs [146]. In an open-label study on pregabalin (ranging from 75 mg/day to 300 mg/day) on 47 patients with both episodic and chronic migraine without overuse of symptomatic drugs, 7 patients had psychiatric comorbidity. A statistically significant reduction in migraine frequency compared to baseline was evident after 1 and 3 months of treatment [147]. Another open-label study demonstrated pregabalin efficacy in treating patients with medication overuse headaches in reducing both mean monthly headache frequency and number of days with medication intake [154].

Gabapentin and pregabalin have demonstrated moderate efficacy in patients with migraine, favorable tolerability profile, and no significant interactions with other drugs. GBP and pregabalin could also represent a valuable alternative for migraine prophylaxis in patients with chronic forms. Nevertheless, the efficacy of pregabalin in migraine requires further double-blind RCTs. The tolerability profile of both drugs is acceptable.

### 3.7 Carbamazepine and Oxcarbazepine

Carbamazepine and oxcarbazepine are tricyclic carbamoyl compounds belonging to the dibenzoazepine family. Carbamazepine activity is mediated by carbamazepine itself and by its active metabolite carbamazepine-10,11-epoxide. Oxcarbazepine is the 10-keto analogue of carbamazepine. A key difference between the two drugs is that oxcarbazepine is not metabolized to an epoxide derivative, leading to a better side-effect profile. Both drugs inhibit voltage-gated sodium channels, increasing the inactivation time in a use-dependent manner. This leads to the inhibition of repetitive neuronal firing, decreased excitatory transmission, and stabilization of the membrane potential in hyperexcitable neurons [155]. Both drugs may also block voltage-gated calcium channels: carbamazepine blocks L-type Ca^2+^ channels, whereas oxcarbazepine inhibits N, P, and R-type Ca^2+^ channels. Moreover, oxcarbazepine enhances potassium currents [155]. Carbamazepine is indicated as monotherapy or adjunctive therapy for treating focal epilepsy, generalized epilepsy, trigeminal neuralgia, and acute manic and mixed episodes in bipolar I disorder [60]. In contrast, oxcarbazepine is indicated as monotherapy or adjunctive therapy for focal epilepsy [60].

Carbamazepine was among the first ASMs to be studied in migraine, but there is only very limited clinical evidence of efficacy [156]. A cross-over, double-blind, placebo-controlled RCT showed that patients treated with carbamazepine had higher responder rates than placebo [156]. So far, no other studies have investigated carbamazepine in migraine prophylaxis; therefore, proof of its efficacy is limited. As a matter of fact, carbamazepine is enlisted as possibly effective for migraine treatment but with a recommendation level C by the latest American Headache Society guidelines [61].

Oxcarbazepine was evaluated for the prophylaxis of episodic migraine in a multicenter, double-blind RCT [157]. Eighty-five patients were randomized to receive oxcarbazepine through titration to a maximum dose of 1200 mg and 85 to placebo for 15 weeks. The trial failed to demonstrate a difference between the oxcarbazepine and placebo groups in the mean change of the number of migraine attacks from baseline during the last 28 days of the double-blind phase. Consequently, oxcarbazepine is classified as possibly or probably ineffective for migraine prophylaxis by the American Headache Society guidelines [61]. A possible hypothesis to explain the lack of efficacy of both drugs in migraine prophylaxis is that their potent, selective inhibitory activity cannot counteract the complex mechanisms underlying migraine pathogenesis, involving also vascular and inflammatory responses [12].

The most common side effects of carbamazepine include dizziness, drowsiness, ataxia, nausea, and vomiting. Other side effects include hyponatremia, which is generally mild and reversible [158], central nervous system depression, hepatotoxicity, renal toxicity, and suicidal ideation [159]. Black box warnings of carbamazepine, although rare, are agranulocytosis, aplastic anemia, and dermatological reactions, including Stevens-Johnson syndrome and toxic epidermal necrolysis [160], which are strongly associated with the HLA-B*15:02 gene in patients with Han Chinese ancestry [161]. Another allele associated with dermatological reactions is HLA-A*31:01, which is present in Japanese, Korean, and European ancestries [162]. Carbamazepine is teratogenic, and its use in women of reproductive age should be limited to cases where its benefits outweigh the increased risk of congenital malformation [159]. Oxcarbazepine has a similar profile of side effects to carbamazepine, although they are less important in frequency and severity [155]. The most common dose-related side effects are fatigue, dizziness, and ataxia. Unlike carbamazepine, there are no black-box warnings for oxcarbazepine. Moreover, among ASMs, oxcarbazepine is one of the drugs with the lowest associated teratogenic effect [163]. Overall, there is very little evidence to support carbamazepine as a prophylactic treatment for migraine, whereas the proof of oxcarbazepine is against its use for migraine treatment. Moreover, their safety and tolerability profile and their possible teratogenicity make these drugs unsuitable for adoption in the prophylaxis of migraine.

### 3.8 Perampanel

Perampanel is a novel antiepileptic drug acting *via* non-competitive antagonism on glutamatergic AMPA receptors and the subsequent inhibition of ion calcium influx [164]. Perampanel has been approved in epilepsy for the adjunctive treatment of focal seizures as well as primary generalized tonic-clonic seizures, both in adults and pediatric patients from 12 years [165, 166], but it does not have indications for migraine or other diseases to date. Pre-clinical studies have shown that perampanel inhibits AMPA-induced calcium influx in isolated rat cortical neurons in a concentration-dependent manner [167, 168]. The antagonists of glutamate receptors might play a role in the treatment of migraine [169], and *in vitro* evidence suggests that perampanel might control pain transmission under conditions of an activated trigeminal system and be able to inhibit, in a concentration-dependent manner, basal CGRP release from isolated rat brainstem [170]. Moreover, various kainate and glutamate receptor antagonists have proven to be effective in animal models of migraine (reviewed in [169]). Glutamate receptors have been localized in areas related to migraine pathophysiology, including the trigeminal ganglion, trigeminal nucleus caudalis, and thalamus [171].

A phase 2 RCT with a placebo was conducted in 206 patients to assess the efficacy and safety of perampanel in migraine prophylaxis (NCT00154063) [172]. The study was completed in 2015, but the results were posted only online and have not been published yet. Briefly, the primary outcome was the change from baseline in migraine period frequency (migraine period was defined as a migraine headache that started, ended, or recurred within 24 hours) per 28 days in the treatment phase. Perampanel was initiated at a dose of 1.0 mg/day for the first 2 weeks, increased to 1.5 mg/day for the next 2 weeks, and then further increased to 2.0 mg/day for 10 weeks. The efficacy analysis was performed on the intention to treat the population, and no differences between placebo (mean -2.94, SD 0.32) and perampanel (mean -2.73, SD 0.39) were reported in the 24h migraine period frequency reduction [172]. Perampanel development for migraine has now been discontinued. A recent small open-label study (28 patients) assessed the effectiveness of perampanel on epileptic seizures. It evaluated its effects per 12 months on migraine attacks in patients with epilepsy and comorbid episodic migraine. Perampanel demonstrated good effectiveness in reducing both epileptic seizures and migraine attacks, as well as an analgesic intake at 6 months, and beneficial effects persisted and remained stable up to the 12-month follow-up in patients with migraine and epilepsy [173]. Regarding tolerability, perampanel can cause dizziness, somnolence, fatigue, irritability, nausea, and falls, as well as hostility and anger, and therefore should probably be avoided or used with caution in patients with significant psychiatric diseases [8, 164]. Overall, there is no adequate evidence to support perampanel as a prophylactic treatment for migraine to date.

### 3.9 Lacosamide

Lacosamide is a third-generation ASM with a unique structure (a functionalized amino acid). It is the only highly selective blocker enhancing the slow inactivation of voltage-gated sodium channels [174]. Indeed, other ASMs such as oxcarbazepine, carbamazepine, and lamotrigine target sodium channels by acting on their fast inactivation (which occurs on a millisecond time scale), whereas lacosamide works on slow inactivation of sodium channels (which appears on a time scale of seconds to minutes) with no effect on fast inactivation [175]. Lacosamide demonstrated anti-hyperalgesic activity in numerous acute and chronic inflammatory and neuropathic pain models but did not have acute antinociceptive activity [176]. In particular, lacosamide reduced trigeminal pain in the rat [177, 178]. Lacosamide has also been found to bind to collapsin response mediator protein-2 (CRMP-2), a cytosolic phosphoprotein mainly expressed in the nervous system and involved in neuronal differentiation, polarization, and axonal outgrowth. Whether and how CRMP-2 relates to the therapeutic action of lacosamide remains to be determined [92, 93]. The use of lacosamide as monotherapy for focal-onset epilepsy has been approved since 2014 but is not approved for migraine or other headaches.

A preclinical study demonstrated that lacosamide negatively modulates CGRP release from rat brainstem explants and inhibits CGRP levels in trigeminal cerebral areas in basal conditions and in the presence of nitroglycerin-mediated stimuli that mimic the activation of pain neurotransmission [177]. Although preliminary clinical data of an open-label study enrolling 22 patients have suggested a positive effect of lacosamide in the prophylaxis of CDH and chronic migraine (reduction in monthly migraine days from 21.4 to 13.0) [179], the results from a double-blind RCT (NCT00440518), including 218 patient and using 100-300 mg of lacosamide, failed to demonstrate any effect on migraine [180]. In particular, the primary outcome of change from baseline in mean migraine headache rates (mean [SD]) was -1.4 (3.12) for placebo, -1.4 (2.68) for the lacosamide 100 mg group, and -1.6 (2.10) for lacosamide 300 mg group [180]. The common side effects of lacosamide include dizziness, fatigue, diplopia, somnolence, drowsiness, and cardiac arrythmias. Skin rashes, hematotoxicity, psychological symptoms, and suicide risk have also been reported [181]. In general, there is no evidence to support lacosamide as a prophylactic treatment for migraine.

### 3.10 Carisbamate

Carisbamate is an alkyl-monocarbamate derivative of felbamate with a broad spectrum of activity. Several potential MOAs have been proposed, including a block of sodium channels, activation of presynaptic Cl^-^ conduction (leading to depression of excitatory neurotransmission), and inhibition of voltage-gated calcium channels, but there is no effect on GABAergic transmission [182]. A comprehensive review of its mechanism is reported elsewhere [182]. Carisbamate showed a broad spectrum of activity in rodent seizure and epilepsy models, including hippocampal and corneal kindling [182, 183]. A phase IIb clinical trial for the treatment of focal onset seizures was completed, demonstrating efficacy and good tolerability of carisbamate, but subsequently failing to demonstrate consistent efficacy across regulatory trials, and the clinical program was discontinued [184]. However, in 2012, carisbamate received an orphan drug designation for treating infantile spasms in West syndrome and is currently in clinical development for Lennox-Gastaut syndrome.

A trial in migraine demonstrated that carisbamate does not have beneficial activity in migraine prophylaxis. A double-blind, placebo RCT enrolled 323 patients using carisbamate at 100, 300, and 600 mg per day *vs*. placebo. In general, carisbamate monotherapy was generally well tolerated, with dizziness and nausea reported more frequently and fatigue, hepatic enzyme increase, and rash less frequently reported [183]. No other studies have been performed to date.

### 3.11 Clonazepam

Clonazepam is a long-acting, high-potency 1,4‐benzodiazepine that has been approved for treating various types of seizures but not for migraines. It is commonly used in some psychiatric disorders (such as panic disorder and other anxiety disorders) and other neurologic diseases (Tourette’s syndrome, movement disorders), or chronic pain syndromes, especially neuropathic pain [185]. For migraine prophylaxis, only one RCT crossover trial was performed in 1979, comparing clonazepam (1 or 2 mg) with a placebo in 38 patients [186]. Both doses of clonazepam were not more effective than the placebo, and more than half of the patients on clonazepam complained of drowsiness [186]. So far, no other RTCs or open-label studies using clonazepam (or any other benzodiazepine) in migraine prophylaxis have been reported. Overall, there is no evidence of the benefit of clonazepam in headache treatment.

### 3.12 Tiagabine

Tiagabine hydrochloride is derived from nipecotic acid, a GABA uptake inhibitor. Tiagabine is a potent and selective presynaptic and glial GABA reuptake inhibitor through the GABA transporter (GAT-1). Tiagabine demonstrated antiallodynic effects in rodent models of neuropathic pain, with the antinociceptive effect related to the inhibition of increased extracellular GABA levels [187]. Tiagabine is approved in Europe and the United States for adjunctive therapy of focal seizures. However, its use is limited by tolerability, including the induction of nonconvulsive status epilepticus and an overall high rate of AEs compared with other ASMs [187, 188]. Tiagabine (4 mg then 8 mg/day) demonstrated efficacy in an open-label clinical trial enrolling 41 patients previously treated with divalproex sodium (discontinued for inefficacy or tolerability) [189]. Thirty-three of 41 patients had at least a 50% reduction in migraine attacks compared to baseline with a mean dose of tiagabine of 10 mg/day. No RCTs have been performed to date. The most prominent side effects are dizziness, asthenia, somnolence, tremor, diarrhea, depression, and emotional lability [187]. At this time, there is not enough evidence to use tiagabine in headache treatment, considering its unfavorable tolerability profile.

### 3.13 Vigabatrin

Vigabatrin is a structural analogue of GABA which irreversibly inhibits GABA-transaminase, increasing GABA transmission in the brain. It is approved for drug-resistant focal epilepsy and treating infantile spasms (West syndrome), particularly with tuberous sclerosis. This drug is now rarely given to adults because of its retinal toxicity and related visual field loss. In migraine, only one study assessed vigabatrin (1000 to 2000 mg/day) compared with a placebo in a 12-week double-blind crossover study in drug-resistant patients [190]. Vigabatrin was not significantly superior to the placebo for headache frequency. Specific AEs were not reported [191]. Overall, the most common treatment-related adverse events were CNS effects beyond the visual field constriction, including drowsiness, dizziness, headache, fatigue, sedation, somnolence, and irritability [192]. Vigabatrin is currently considered not effective in migraine.

### 3.14 Acetazolamide

Acetazolamide is a sulfonamide derivative acting as a carbonic anhydrase inhibitor with effectiveness in preventing episodic ataxia type 2 attacks [193] and with some beneficial effects in managing periodic paralysis, myotonia, and para-myotonia congenita [194]. A beneficial prophylactic effect of acetazolamide has also been reported in a few cases of familial hemiplegic migraine [195]. FDA-approved indications include glaucoma, idiopathic intracranial hypertension, congestive heart failure, altitude sickness, periodic paralysis, and epilepsy. In this latter case, the inhibition of carbonic anhydrase (extensively expressed in the CNS) seems to retard abnormal, excessive, paroxysmal electrical discharge from neurons. In a multicenter RCT of 12 weeks, daily oral 500 mg acetazolamide and placebo were administered in patients with migraine with or without aura [196]. The study was prematurely stopped because of many withdrawals in enrolled patients (27 in the placebo group and 26 in the acetazolamide group). Acetazolamide was poorly tolerated in an oral dose of 500 mg with no effect on migraine [196]. The commonly reported AEs were fatigue, nausea, vomiting, abdominal pain, and diarrhea. Other studies reported paresthesias, dysgeusia, and polyuria. The risk for paresthesias and dysgeusia increased with higher ACZ doses [197]. Therefore, acetazolamide is considered ineffective in migraines, and no other studies have been performed.

## 4 ONGOING CLINICAL TRIALS

Only two RCTs are ongoing with ASMs in migraine [198, 199]. In particular, both studies investigate the effect of topiramate extended-release compared with placebo in pediatric patients (6 to 11 years) with migraine (with or without aura) [198, 199]. As expected, ongoing or active RCTs are substantially investigating the novel anti-CGRP drugs, monoclonal antibodies, and gepants recently reviewed for the pediatric population in [200] and adults in [201].

## CONCLUSION

Topiramate and divalproex sodium/sodium valproate are the only approved ASMs for migraine prevention as they are supported by high-quality evidence (namely double-blind RTCs). No benefit in migraine prophylaxis has been reported for perampanel, lacosamide, carisbamate, clonazepam, oxcarbazepine, tiagabine, or vigabatrin. Other ASMs have demonstrated some benefits in migraine prevention (or at least in migraine with aura). These include lamotrigine, zonisamide, gabapentin, pregabalin, carbamazepine, and levetiracetam, but none are indicated for migraine/headache, and further evidence is needed. Along with effectiveness, tolerability should be carefully considered in prescribing ASMs in migraine, above all for drugs with known or potential teratogenicity risk, bearing in mind that migraines are most prevalent in women of childbearing potential.

Several new ASMs have been approved for epilepsy in recent years [8], and numerous have been tested, providing novel potential drugs for non-epileptic conditions (including migraine) to enrich the treatment armamentarium and anti-CGRP drugs. To note, only one study was performed addressing the safety and efficacy of anti-CGRP mAbs compared to ASMs, demonstrating that erenumab had a better safety and efficacy profile than topiramate (HER-MES study) [81].

Anti-CGRP mAbs but not ASMs are usually restricted by different reimbursement conditions (including prior preventives failure) in several countries, holding ASMs as prescribed before anti-CGRP drugs. Nevertheless, the latest European guidelines on anti-CGRP mAbs [202] suggest a first-line use for these drugs based on their effectiveness and tolerability profile. Further studies comparing anti-CGRP mAbs and current preventive treatments are required to strengthen their use in naïve patients to preventive treatments. Continuing investigation of putative MOAs of ASMs in migraine would be useful to understand its pathophysiology better, identify novel potential neuronal or non-neuronal targets, and optimize treatment.

## Figures and Tables

**Fig. (2):**
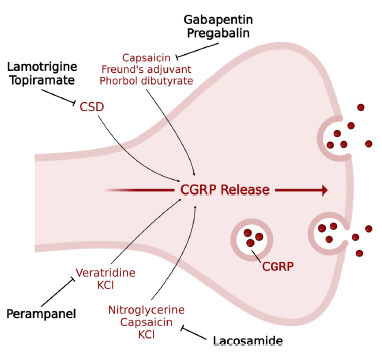
Putative modulation of basal and induced release of CGRP by ASMs. The reported stimuli of CGRP release are those specifically used/investigated in studies involving ASMs. ASMs inhibit CGRP release and do not affect inducing stimuli. **Abbreviations**: CGRP, calcitonin gene-related protein; CSD, cortical spread depression.

**Table 1:** Indicative doses and common reported adverse events for adults in migraine and epilepsy of antiseizure medications assessed for migraine preventive treatment.

**Antiseizure ** **Medications**	Doses in Migraine (mg/die)*	Doses in Epilepsy (mg/die)*	**Common Reported Adverse Events**
Topiramate^#^	50-200	200-400	Paresthesia, anorexia, weight loss, altered taste, memory impairment, fatigue, insomnia, nausea, diarrhea, somnolence, dizziness.
Valproate^#^	500-2000	600-2500	Fatigue, weight gain, dizziness, nausea and tremor. Risk of congenital malformations and neurodevelopmental disorders.
Lamotrigine	50-300*^§^*	200-400	Cutaneous adverse events, dizziness, vertigo, sleep disturbances, weight increase and asthenia.
Zonisamide	100-400*^§^*	200-600	Fatigue, paresthesia, kidney stones, memory impairment, decreased hemoglobin level, and weight loss.
Gabapentin	900-3000	900-3600	Asthenia, nausea, somnolence, confusion, dizziness, infections, ataxia, weight gain, blurred vision, drowsiness, abnormal thinking and constipation.
Pregabalin	75-300*^§^*	150-600	Asthenia, nausea, somnolence, confusion, dizziness, infections, ataxia, weight gain, blurred vision, drowsiness, abnormal thinking and constipation.
Perampanel	2.0*^§^*	4-12	Dizziness, somnolence, fatigue, irritability, nausea, and falls.
Levetiracetam	1000-3000	1000-3000	Somnolence, dizziness, irritability, anxiety, weight gain, depression, asthenia, memory problems and lack of concentration.
Carbamazepine	400-1200*^§^*	400-1200	Dizziness, drowsiness, ataxia, nausea, and vomiting.
Oxcarbazepine	600-1200*^§^*	600-1200	Dizziness, drowsiness, ataxia, nausea, and vomiting.
Lacosamide	100-300*^§^*	200-400	Dizziness, fatigue, diplopia, somnolence, drowsiness, skin rashes, and cardiovascular abnormalities.
Vigabatrin	1000-2000*^§^*	1000-3000	Drowsiness, dizziness, fatigue, sedation, somnolence, irritability, visual field constriction.
Carisbamate	100-600*^§^*	300-1600	Dizziness, nausea, fatigue, hepatic enzyme increase, and rash.
Tiagabine	4-10*^§^*	4-32	Dizziness, asthenia, somnolence, tremor, diarrhea, depression and emotional lability.
Clonazepam	1-2*^§^*	0.5-4	Tiredness, dizziness, unsteadiness, impaired attention and memory, irritability, nausea, loss of appetite.
Acetazolamide	500*^§^*	8-30 (per kg)	Fatigue, nausea, vomiting, abdominal pain, diarrhea, paresthesias, dysgeusia and polyuria.

**Note:** *Doses can significantly change basing on concomitant medications, indication, and patient age, weight and comorbidities. Reported interval for epilepsy and migraine are the indicative usual dose and/or doses used in clinical trials; ^#^Approved for migraine; ^§^Only one randomized clinical trial.

## LIST OF ABBREVIATIONS

ASMs: Antiseizure Medications
BDNF: Brain-derived Neurotrophic Factor
CDH: Chronic Daily Headache
CGRP: Calcitonin Gene-related Peptide
CNS: Central Nervous System
CSD: Cortical Spreading Depression
MOA: Mechanisms of Action
PSNs: Primary Sensory Neurons
TG: Trigeminal Ganglia
